# Gastric Distension Index: A Novel Radiographic Marker Associated With Postoperative Gastric Stasis After Gastrectomy

**DOI:** 10.1002/ags3.70077

**Published:** 2025-08-11

**Authors:** Hiroki Harada, Yoshiko Yamaoka, Akiko Watanabe, Kota Okuno, Shohei Fujita, Mikiko Sakuraya, Tadashi Higuchi, Koshi Kumagai, Keishi Yamashita, Naoki Hiki

**Affiliations:** ^1^ Department of Upper Gastrointestinal Surgery Kitasato University School of Medicine Sagamihara Kanagawa Japan; ^2^ Department of Lower Gastrointestinal Surgery Kitasato University School of Medicine Sagamihara Kanagawa Japan; ^3^ Division of Advanced Surgical Oncology, Department of Research and Development Center for New Medical Frontiers Kitasato University School of Medicine Sagamihara Kanagawa Japan

**Keywords:** gastrectomy, gastric cancer, gastric dilatation, index, stasis

## Abstract

**Background:**

Gastric remnant distension after gastrectomy is associated with gastrointestinal symptoms and poor postoperative outcomes, particularly in elderly patients. However, no objective clinical index has been established to define or predict this condition.

**Methods:**

We retrospectively analyzed 67 patients who underwent distal gastrectomy (DG) in 2019 with upright abdominal X‐ray imaging. Radiographic indices were evaluated for their association with gastrointestinal symptoms and the need for intervention for postoperative gastric stasis. The maximum width of the gastric bubble was the most predictive and standardized into the Hiki Index (HI). A cutoff value was determined from ROC analysis based on stasis‐related intervention. Validation was performed in a prospectively collected cohort of 136 patients who underwent DG or pylorus‐preserving gastrectomy (PPG) between 2022 and 2023 after clinical implementation of the HI.

**Results:**

In the test set, HI showed high predictive accuracy for stasis‐related intervention (AUC 0.78, sensitivity 85.7%, specificity 76.7%). In the validation set, HI predicted symptoms such as nausea (AUC 0.77), bloating (0.77), and belching (0.90). HI‐based gastric distension was not associated with postoperative complications. Age < 75 years (OR 3.29), female sex (OR 2.58), and undergoing PPG (OR 6.21) were identified as independent risk factors for gastric distension.

**Conclusion:**

The HI is a reproducible and practical radiographic indicator for identifying patients at risk of postoperative gastric stasis and related symptoms. Its use may facilitate early intervention and improve postoperative care. Prospective implementation of the HI contributed to more consistent symptom documentation and clearer associations between HI and gastrointestinal symptoms.

## Graphical abstract

This study introduces the Hiki Index (HI), a novel radiographic metric based on upright abdominal X‐rays, to objectively assess postoperative gastric dilatation after gastrectomy. The HI demonstrated strong predictive value for gastrointestinal symptoms and the need for therapeutic intervention, offering a practical tool for early detection and management of gastric stasis.

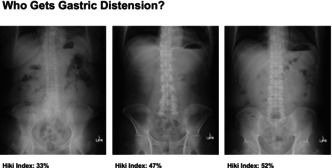

## 1 Introduction

Gastric cancer remains a malignancy with a high incidence and mortality rate not only in Japan but also worldwide, and surgical resection continues to play a critical role as a curative treatment [1, 2, 3]. In Japan, the widespread implementation of gastric cancer screening programs has contributed to early detection and improved surgical outcomes [4]. However, with the increasing aging population, the proportion of elderly patients undergoing gastrectomy is rising. In these patients, pre‐existing comorbidities and declining physical resilience pose significant challenges for surgical decision‐making and postoperative management. Careful preoperative assessment and appropriate perioperative management are essential for improving outcomes in elderly patients undergoing gastrectomy.

One of the significant postoperative complications is gastric remnant distension. This condition arises from various factors, including impaired gastric peristalsis, anastomotic stenosis caused by edema, ulceration, or scar formation [5]. Gastric remnant distension can result in inadequate emptying of gastric contents, leading to symptoms such as nausea, vomiting, and bloating, and increasing the risk of aspiration pneumonia due to reflux of gastric contents [6]. In elderly patients, age‐related decline in swallowing function and weakened immune responses further exacerbate these risks [7]. Aspiration pneumonia not only contributes to increased postoperative mortality and prolonged hospital stays but also significantly diminishes patients' quality of life [8].

Early diagnosis of gastric remnant distension is crucial for preventing the onset and progression of these complications. Timely implementation of gastric decompression, fasting with intravenous fluids, and pharmacological intervention can help reduce the risk of aspiration pneumonia and prevent delays in postoperative recovery. Additionally, early diagnosis may prevent prolonged severe gastric distension, which could otherwise lead to impaired bowel function and systemic deterioration. Therefore, establishing an objective and reproducible diagnostic indicator for gastric remnant distension is a critical aspect of improving postoperative care.

However, no standardized clinical indicators for defining gastric remnant distension have been established to date. Recently, Watanabe et al. proposed a radiographic marker, now referred to as the “Hiki Index (HI),” to objectively assess gastric distension after gastrectomy at our institution, using the same time period as our test cohort but with different exclusion criteria [9].

This study aimed to identify clinically useful radiographic indicators of pathological gastric dilatation that require therapeutic intervention—such as fasting with intravenous fluids, gastric decompression, or pharmacologic treatment—based on multiple parameters obtained from plain abdominal radiograph (X‐ray), and to evaluate their clinical utility.

## 2 Patients and Methods

### 2.1 Patients

**FIGURE 1:**
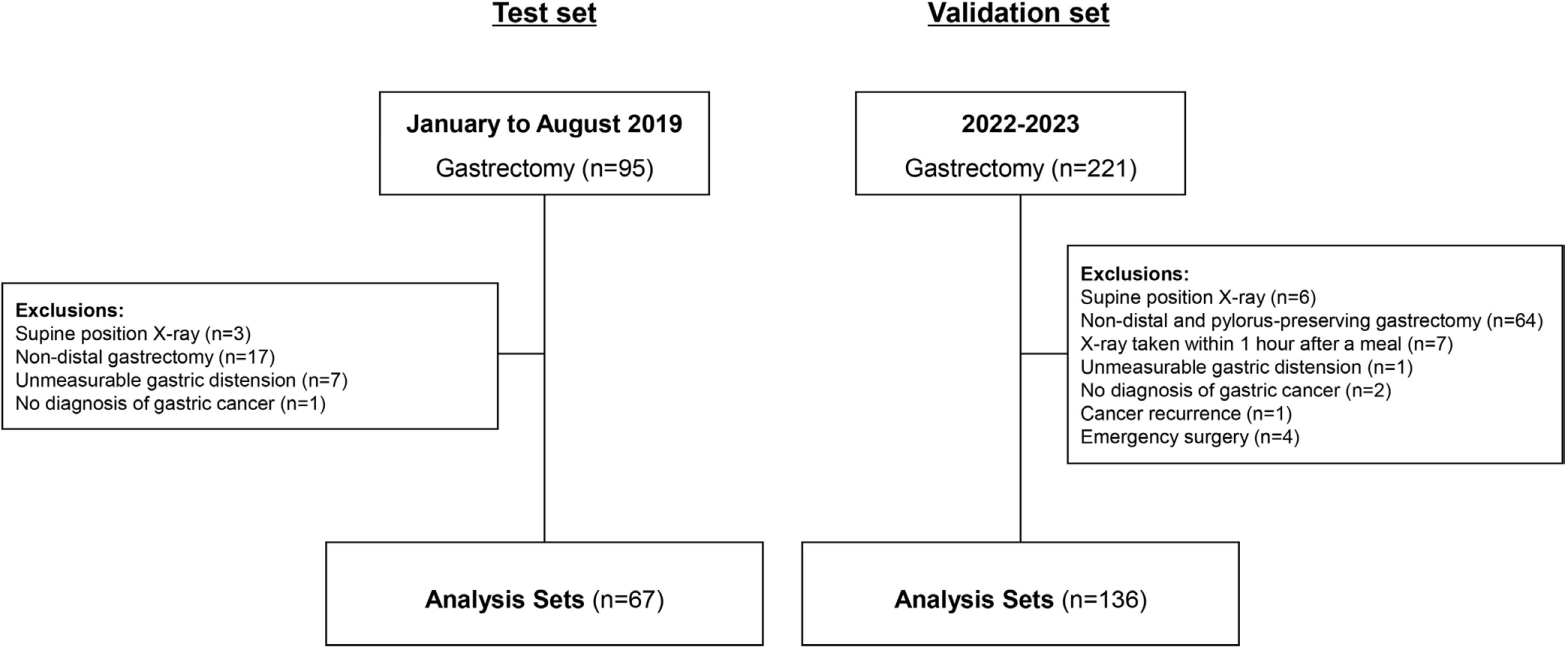
Flow diagram of patient selection and study design.

This retrospective study included patients who underwent distal gastrectomy (DG) at Kitasato University Hospital between January and August 2019. Of the patients included, patients who underwent postoperative plain abdominal X‐rays in an upright position were included in the final analysis (Figure 1). Based on these findings, a definition of gastric dilatation was established, and therapeutic interventions were implemented for applicable patients from 2022 onward. To validate the reliability of the study results, an additional analysis was conducted in a validation set comprising patients who underwent DG or pylorus‐preserving gastrectomy (PPG) between 2022 and 2023, examining the association between gastric dilatation and the presence of gastrointestinal symptoms (Figure 1). This validation set was prospectively collected after the clinical implementation of the HI in 2022. Importantly, when gastric distension was identified based on a cutoff value derived from receiver operating characteristic (ROC) analysis in the test set, patients underwent clinical evaluation and received therapeutic interventions such as fasting or pharmacologic treatment, regardless of the presence or absence of gastrointestinal symptoms. This ensured more standardized and proactive management and documentation in real‐world practice. Although the time frame and institution overlap with a previous study the exclusion criteria differed, resulting in a distinct patient population [9]. The present study was approved by the ethics committee of the Kitasato University School of Medicine (B19‐280).

### 2.2 Surgical Technique

Gastrectomy was performed in patients without distant metastases, such as peritoneal dissemination. In DG, the celiac and hepatic branches of the vagus nerve were not routinely preserved, following standard oncologic procedures involving D1+ or D2 lymph node dissection. In contrast, during PPG, special attention was paid to preserve the hepatic branch of the vagus nerve and the pyloric cuff, according to established techniques aimed at maintaining pyloric function and promoting physiological gastric emptying.

In Billroth‐I reconstruction, a side‐to‐side gastroduodenostomy was performed using a linear stapler, with the anastomotic site selected to ensure a tension‐free and well‐vascularized connection. In Roux‐en‐Y reconstruction, the jejunum was transected approximately 20 cm distal to the ligament of Treitz. A stapled gastrojejunostomy was created in an antecolic manner, followed by a side‐to‐side jejunojejunostomy approximately 45 cm distal to the gastrojejunostomy. In PPG, gastrogastrostomy was performed using the Piercing method with a linear stapler [10]. A small entry hole was created on the lesser curvature of the anal‐side remnant stomach, and a side‐to‐side anastomosis was constructed with the oral‐side remnant stomach. These procedures were standardized across surgeons to minimize variability in anastomotic configuration.

### 2.3 Abdominal Simple X‐Ray Protocol

Postoperative upright abdominal plain X‐ray was performed following a standardized protocol. To account for potential variation in gastric content due to postoperative day and the timing of meals, our institution employs a standardized clinical pathway for abdominal radiography. On postoperative day 1 (POD1), supine abdominal X‐rays are taken to assess the immediate postoperative status. From POD3 onward, imaging is performed in the upright position to more accurately evaluate the natural gravitational descent of gastric contents and the degree of gastric distension. There were no restrictions regarding the timing of imaging. However, in patients presenting with symptoms such as nausea, vomiting, bloating, or belching X‐ray images taken at the time of symptom onset were prioritized for analysis.

In the validation set, radiographs were obtained at least 1 h after meals to minimize the influence of recent food intake, allowing for a relatively consistent assessment of gastric contents across patients. This partially standardized timing aimed to reduce inter‐patient variability and improve reproducibility in evaluating the HI.

### 2.4 X‐Ray Measurement Parameters

**FIGURE 2:**
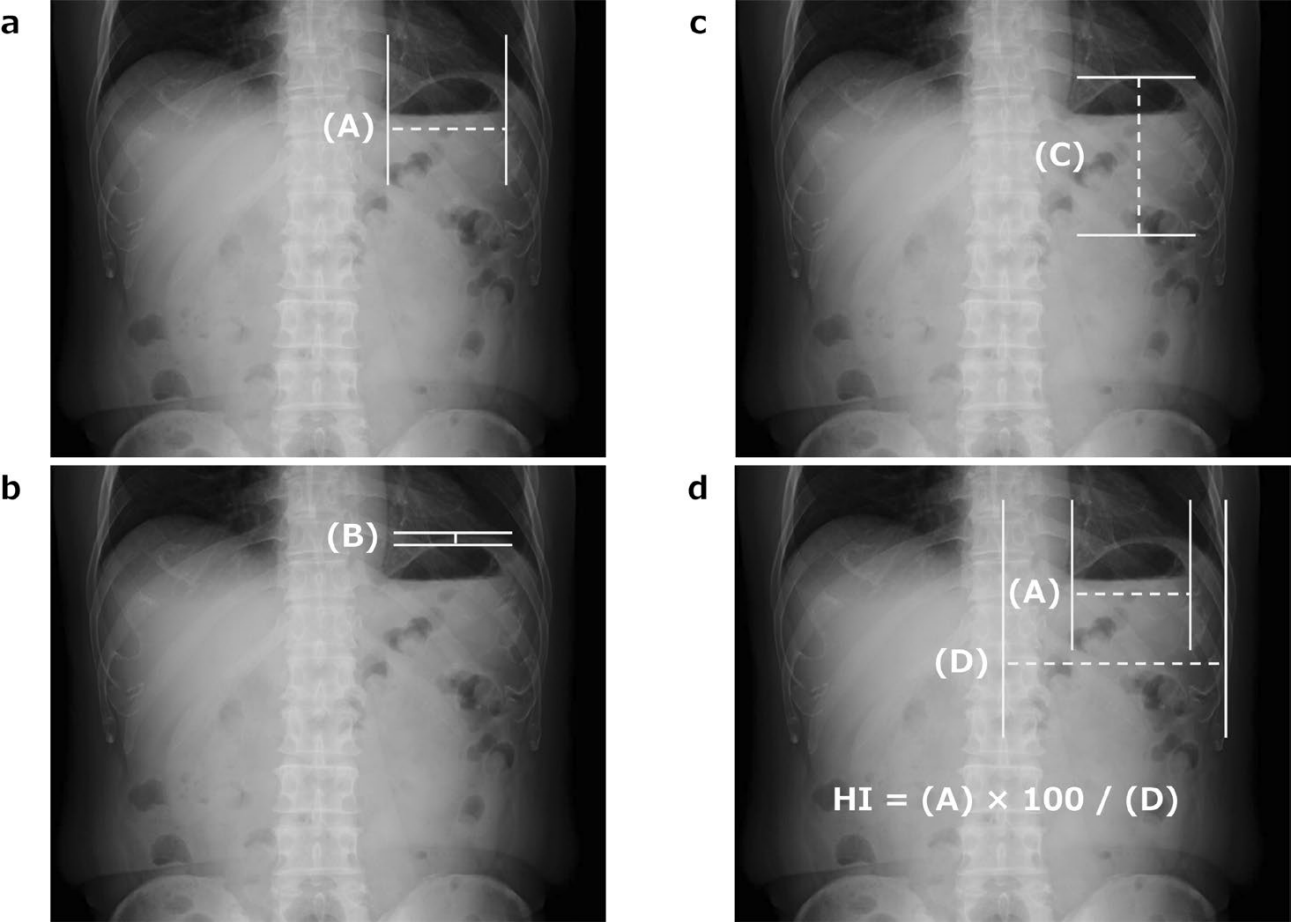
Radiographic measurements used to assess postoperative gastric distension. (a) Maximum horizontal width of the gastric bubble. (b) Vertical distance from the apex of the gastric bubble to the left diaphragm. (c) Head‐to‐tail (longitudinal) length of the remnant stomach. (d) Reference distance from the center of the vertebral body to the left costophrenic angle. The Hiki Index (HI) was calculated as the ratio of each measurement (a) to (d), expressed as a percentage.

The following radiographic parameters, potentially associated with gastric remnant distension, were measured on abdominal plain X‐ray images:

(A) Maximum width of the gastric bubble (Figure 2a), (B) distance between the left diaphragm and the apex of the gastric bubble (Figure 2b), (C) vertical length of the stomach (Figure 2c), furthermore, to account for differences in body habitus, the distance was divided by the length from the center of the vertebral body to the left costophrenic angle (D), and the resulting ratio was expressed as a percentage.

The Hiki Index (HI) = (A) × 100/(D) (Figure 2d).

The relationship between these X‐ray measurements and the presence or absence of gastrointestinal symptoms (nausea, vomiting, bloating, and belching) and the need for therapeutic intervention was evaluated using ROC curves analysis. The parameter with the highest area under the curve (AUC) was identified as the most relevant indicator.

### 2.5 Criteria for Therapeutic Intervention in Patients With Gastrointestinal Symptoms

At our institution, oral intake typically begins on postoperative Day 2. Patients who underwent fasting with intravenous therapy from postoperative Day 2 onward due to impaired oral intake caused by gastric content stasis, as well as those who required pharmacological treatment or nasogastric tube insertion, were defined as patients requiring therapeutic intervention.

The test set was a retrospective study, and therapeutic interventions were initiated at the discretion of the attending physician based on a comprehensive assessment of clinical symptoms (e.g., nausea, vomiting, bloating, belching), physical findings, and persistent radiographic evidence of gastric dilatation on plain abdominal X‐rays. Pharmacological treatments included prokinetic agents such as mosapride and metoclopramide, the macrolide antibiotic erythromycin, and the Japanese herbal medicine rikkunshito. Notably, the clinical indication for intervention was not based on standardized diagnostic criteria for gastric stasis, and objective evaluations such as gastric emptying scintigraphy were not routinely performed. Therefore, the diagnosis of gastric dysfunction and the decision to initiate treatment in this study were based on pragmatic clinical judgment in routine practice rather than formal diagnostic standards.

### 2.6 Validation Analysis

In the analysis of the validation set, the association between gastric dilatation, as defined by the HI, and postoperative gastrointestinal symptoms (nausea, vomiting, bloating, and belching) was evaluated using receiver operating characteristic ROC curve analysis. Sensitivity, specificity, and AUC values were compared to verify whether the cutoff value derived from the initial cohort could be consistently applied to the validation set.

Additionally, the reproducibility and stability of the selected indicator were examined across different patient backgrounds. As the validation set reflects a more prospective cohort following routine clinical recognition of HI, the results may reflect improved data quality and more accurate capture of gastrointestinal symptoms. The analysis further evaluated whether these patient‐specific factors influenced the predictive accuracy of the identified indicator. Finally, the relationship between the selected indicator and postoperative complications—including anastomotic leakage, ileus, pancreatic fistula, intra‐abdominal abscess, and wound infection—was analyzed to determine the broader clinical applicability and reliability of the indicator.

### 2.7 Patient Background and Clinical Variables

Baseline patient characteristics, including age, sex, comorbidities, and body mass index (BMI), were collected. Tumor‐related pathological factors, such as tumor size, location, and clinical‐pathological stage, were also recorded. Additionally, surgical factors, including procedure type, operative time, blood loss, duration of postoperative hospitalization, and postoperative complications, were evaluated. The severity of postoperative complications was classified according to the Clavien‐Dindo classification system [11].

### 2.8 Statistical Analysis

Continuous variables were analyzed using the student's *t*‐test, while categorical variables were assessed using the Mann–Whitney *U* test or Chi‐square test, as appropriate. A *p*‐value < 0.05 was considered statistically significant. ROC curve analysis was performed to calculate the AUC, and the optimal cutoff value was determined. Associations between patient characteristics, surgical factors, and postoperative outcomes were examined using a logistic regression model, with odds ratios (OR) and 95% confidence intervals (CI) calculated. Statistical analysis was conducted using SAS software (JMP Pro 16, SAS Institute, Cary, NC, USA).

## 3 Results

### 3.1 Patient Background

The study population was divided into two groups: patients who developed postoperative gastrointestinal symptoms, including nausea, vomiting, bloating, belching, or requiring therapeutic intervention (*n* = 14), and those who did not present these symptoms (*n* = 53). There were no significant differences between the two groups in terms of sex, age, BMI, preoperative serum albumin levels, prevalence of diabetes mellitus, or surgical approach, including open, laparoscopic, or robot‐assisted surgery. However, the proportion of patients with American Society of Anesthesiologists Physical Status (ASA‐PS) grade III was significantly higher in the symptomatic group (*p* = 0.0182), suggesting that poor preoperative physical status may be associated with postoperative gastrointestinal symptoms. Similarly, no significant differences were observed regarding the extent of lymph node dissection, method of reconstruction, or tumor stage. The length of hospital stay was significantly longer in the group with gastrointestinal symptoms (*p* = 0.0110) (Table 1).

**TABLE 1:** Patient characteristics with and without gastrointestinal symptoms after distal gastrectomy.

Variables	Postoperative gastrointestinal symptoms		*p*
	Negative (*n* = 53)	Positive (*n* = 14)	
Age at surgery, years, median (range)	71 (45–88)	74.5 (46–84)	0.5100
Sex			
Male/Female	39/14	10/4	0.8714
Body mass index, kg/m2, median (range)	21.4 (15.7–27.5)	21.6 (17.8–28.4)	0.9503
ASA‐PS			
Grade I/II/III	3/47/3	2/8/4	0.0182
Diabetes mellitus			
Yes/No	10/43	2/12	0.6909
Preoperative albumin, g/dL, median (range)	4.2 (2.6–5.2)	4.1 (2.9–4.5)	0.2465
Approach for gastrectomy			
Open/Laparoscopy/Robot	8/38/7	2/9/3	0.7434
Lymph node dissection			
D1+/D2	35/18	7/7	0.2698
Anastomotic procedures			
Billroth‐I/Roux‐en‐Y	26/27	8/6	0.5904
Tumor location			
Upper/Middle/Lower	5/25/23	0/7/7	0.4850
Tumor diameter, mm, median (range)	33 (4–120)	32.5 (5.7–70)	0.9495
Pathological depth of invasion (pT)			
pT1a/1b/2/3/4a/X	15/19/6/6/7	4/8/1/1/0	0.4831
Pathological lymph node metastasis (pN)			
pN0/1/2/3a/3b	42/4/2/2/3	10/3/0/0/1	0.5206
Postoperative hospital stay, days, median (range)	8 (5–35)	12 (8–57)	0.0110

Abbreviation: ASA‐PS American Society of Anesthesiologists Physical Status.

### 3.2 Definition of Indices Associated With Gastric Distension

Comparison of the AUC for radiographic indices associated with gastrointestinal symptoms (nausea, vomiting, abdominal bloating, belching) and the necessity for therapeutic intervention after gastrectomy revealed that Index A exhibited the highest predictive accuracy, followed closely by Index B. AUC values for gastrointestinal symptoms using Index A were 0.54 for nausea, 0.62 for vomiting, 0.60 for abdominal bloating, and 0.69 for belching (Table 2). In particular, for predicting the necessity for therapeutic intervention, Index A demonstrated excellent predictive performance, with an AUC of 0.78, sensitivity of 85.7%, and specificity of 76.7% (*p* = 0.0044) (Table 2). Although Index B also showed high predictive accuracy, Index A was adopted for further refinement due to its slightly superior performance. Furthermore, in terms of detection rate, Index A demonstrated the highest value at 100% (*n* = 67), compared to Index B at 97% (*n* = 65) and Index C at 77.6% (*n* = 52).

**TABLE 2:** Diagnostic accuracy of radiographic indices for predicting gastrointestinal symptoms and therapeutic intervention.

Variables (*n* = 67)	Cutoff value	AUC	Sensitivitiy (%)	Specificity (%)	*p*
(A) Maximum width of the gastric bubble (*n* = 67)					
Nausea	71 mm	0.54	50.0	72.1	0.7182
Vomiting	71 mm	0.62	66.7	71.9	0.4497
Bloating	63 mm	0.60	58.3	65.5	0.1624
Belching	73 mm	0.69	66.7	81.3	0.3571
Therapeutic intervention	71 mm	0.78	85.7	76.7	0.0044
(B) Distance between the left diaphragm and the apex of the gastric bubble (*n* = 65)					
Nausea	4.4 mm	0.68	83.3	59.3	0.8525
Vomiting	4.4 mm	0.71	100	58.1	0.1859
Bloating	4.3 mm	0.59	66.7	62.3	0.8042
Belching	2.6 mm	0.56	33.3	90.3	0.5678
Therapeutic intervention	4.4 mm	0.77	100	62.1	0.0088
(C) Vertical length of the stomach (*n* = 52)					
Nausea	100 mm	0.54	50.0	72.9	0.7659
Vomiting	81 mm	0.57	100	40.0	0.8944
Bloating	93 mm	0.62	66.7	67.4	0.2487
Belching	95 mm	0.72	100	66.0	0.5212
Therapeutic intervention	95 mm	0.70	75.0	66.6	0.2139
The Hiki index (*n* = 67)					
Nausea	52.5%	0.64	50.0	85.3	0.2478
Vomiting	60.2%	0.66	66.7	95.3	0.2380
Bloating	42.6%	0.64	75.0	58.2	0.1261
Belching	52.5%	0.67	66.7	84.4	0.2782
Therapeutic intervention	51.0%	0.77	71.4	83.4	0.0062

Abbreviation: AUC, area under the curve.

### 3.3 Cutoff Value for Gastric Distension as a Clinical Index

By standardizing indicator, A as a percentage by dividing it by the distance from the center of the vertebral body to the left diaphragmatic angle, it became possible to achieve a uniform assessment that accounts for individual differences in body shape (defined as the HI) (Figure 2d). ROC curve analysis using the HI demonstrated high predictive accuracy and specificity for each symptom and therapeutic intervention, including nausea (AUC 0.64, specificity 85.3%), vomiting (AUC 0.66, specificity 95.3%), bloating (AUC 0.64, specificity 58.2%), belching (AUC 0.67, specificity 84.4%), and the need for therapeutic intervention (AUC 0.77, specificity 83.4%) (Table 2).

### 3.4 Association Between Preoperative Patient Characteristics and HI

We evaluated whether preoperative patient characteristics, including serum albumin level, diabetes mellitus, and ASA‐PS, were associated with the degree of postoperative gastric dilatation, as quantified by the HI. As shown in Table S1, there were no statistically significant differences in the HI when stratified by albumin level (≥ 3.8 vs. < 3.8 g/dL, *p* = 0.7031), diabetes mellitus status (*p* = 0.4907), or ASA‐PS grade (*p* = 0.1013).

### 3.5 Association Between Gastric Distension and Gastrointestinal Symptoms in the Validation Set

In the validation set, 136 patients who underwent DG (*n* = 122) or PPG (*n* = 14) between 2022 and 2023 were analyzed. This cohort represents a prospectively collected dataset from clinical practice after implementation of HI‐based assessment. In the validation set, we performed ROC curve analysis using the HI to evaluate its ability to predict gastrointestinal symptoms. For nausea, the HI showed an AUC of 0.77, with a sensitivity of 84.2% and specificity of 67.5% (cutoff: 48.8%). For bloating, the HI demonstrated similar accuracy (AUC 0.77), with a sensitivity of 70.4% and specificity of 82.6% (cutoff: 52.2%). Belching showed the highest predictive performance, with an AUC of 0.90, 100% sensitivity, and 79.4% specificity (cutoff: 53.0%). In contrast, vomiting showed lower predictive performance (AUC 0.60, sensitivity 50.0%, specificity 78.9%) and did not reach statistical significance (*p* = 0.2253) (Table 3).

**TABLE 3:** ROC curve analysis for gastrointestinal symptoms using the Hiki Index (HI) in the validation cohort.

Variables	Cutoff value	AUC	Sensitivitiy (%)	Specificity (%)	*p*
Nausea	48.8%	0.77	84.2	67.5	0.0005
Vomiting	54.1%	0.60	50.0	78.9	0.2253
Bloating	52.2%	0.77	70.4	82.6	< 0.0001
Belching	53.0%	0.90	100	79.4	< 0.0001

Abbreviation: AUC, area under the curve.

### 3.6 Associations of Gastric Distension (HI ≥ 51%) With Postoperative Outcomes

Table 4 summarizes the relationship between gastric distension (HI ≥ 51%) and postoperative complications. Among the 136 patients in the validation cohort, 42 (30.9%) were classified as having gastric distension. There were no significant differences observed between patients with gastric distension and those without regarding anastomotic leakage (*p* = 0.8912), ileus (*p* = 0.8919), pancreatic fistula (*p* = 0.6530), intra‐abdominal abscess (*p* = 0.2417), or wound infection (*p* = 0.8943) (Table 4).

**TABLE 4:** Postoperative complications stratified by the presence or absence of gastric distension (HI ≥ 51%).

Variables	The Hiki index (HI)		*p*
	Non‐distention (*n* = 94)	Distention (*n* = 42)	
Postoperative complications			
Anastomotic leakage	5	2	0.8912
Ileus	5	2	0.8919
Pancreatic fistula	3	2	0.6530
Intra‐abdominal abscess	3	0	0.2417
Wound infection	4	2	0.8943

### 3.7 Associated Factors for Gastric Distension (HI ≥ 51%) in the Validation Set

Factors associated with gastric distension (HI ≥ 51%) were evaluated using univariate and multivariate analyses. In the multivariate analysis, age < 75 years (OR 3.29, 95% CI 1.24–8.75, *p* = 0.0171), female sex (OR 2.58, 95% CI 1.06–6.27, *p* = 0.0361), and undergoing PPG (OR 6.21, 95% CI 1.36–28.33, *p* = 0.0184) were identified as independent risk factors for postoperative gastric distension (Table 5).

**TABLE 5:** Univariate and multivariate analyses of factors associated with high Hiki Index (HI ≥ 51%).

Variables	Univariate analysis		Multivariate analysis	
	Odds ratio (95% CI)	*p*	Odds ratio (95% CI)	*p*
Age at surgery, years				
< 75/≥ 75	2.82 (1.28–6.64)	0.0097	3.29 (1.24–8.75)	0.0171
Sex				
Female/Male	2.58 (1.23–5.51)	0.0121	2.58 (1.06–6.27)	0.0361
Body mass index, kg/m2				
> 25/≤ 25	0.47 (0.15–1.26)	0.1388	0.39 (0.12–1.26)	0.114
Tumor location				
Upper or Middle/Lower	1.12 (0.53–2.42)	0.7733	0.78 (0.29–2.11)	0.6286
Tumor diameter, mm				
> 33/≤ 33	1.44 (0.69–3.00)	0.3309	1.08 (0.39–3.16)	0.8883
Hiatus hernia				
Yes/No	0.37 (0.13–0.93)	0.0339	0.51 (0.17–1.57)	0.2405
Approach for gastrectomy				
Open/Laparoscopy or Robot	2.06 (0.77–5.43)	0.1495	1.98 (0.48–8.23)	0.3479
Type of gastrectomy				
PPG/DG	3.45 (1.12–11.19)	0.0312	6.21 (1.36–28.33)	0.0184
Lymph node dissection				
D2/D1+	1.54 (0.74–3.22)	0.2477	1.85 (0.59–5.77)	0.2881
Anastomotic procedures				
Roux‐en‐Y/Billroth‐I or Gastrogastrostomy	1.56 (0.75–3.28)	0.2290	2.48 (0.83–7.37)	0.1035
Operation time, min				
> 397/≤ 397	1.52 (0.73–3.19)	0.2648	1.04 (0.41–2.64)	0.9323
Blood loss, g				
> 68.5/≤ 68.5	1.00 (0.48–2.08)	1	0.75 (0.28–2.04)	0.5785

Abbreviations: CI, confidence interval; DG, distal gastrectomy; PPG, pylorus‐preserving gastrectomy.

## 4 Discussion

Postoperative gastric dilatation is a common and clinically important condition that can lead to symptoms such as nausea, vomiting, abdominal bloating, and poor oral intake. While plain abdominal radiographs often reveal signs of gastric content retention in these patients, there is currently no standardized or objective definition of gastric dilatation specific to the remnant stomach after gastrectomy. The lack of a reproducible radiological index limits early diagnosis and appropriate intervention in postoperative management.

In this study, we developed and validated a novel radiographic metric—the Hiki index (HI)—derived from upright abdominal X‐ray images, to objectively assess gastric dilatation after gastrectomy. The HI is based on the maximum width of the gastric bubble (Index A), corrected for body morphology, and was shown to be a highly reliable and clinically relevant indicator of gastric stasis and related symptoms.

Three major findings support the utility of the HI. First, Index A, representing the maximum width of the gastric bubble, demonstrated the strongest association with gastrointestinal symptoms and the need for therapeutic intervention among all indices examined. This is anatomically plausible, as postoperative gastric dilatation most commonly presents in the upper part of the remnant stomach, corresponding to the gastric bubble. Previous studies have described the clinical impact of gastric tube distension after esophagectomy, including increased risk of aspiration pneumonia and respiratory complications, but no such objective indicator has been validated in the context of gastrectomy [12]. In our analysis, Index A showed excellent predictive ability, particularly in cases requiring intervention, supporting its use as a simple and effective screening marker.

Second, to account for anatomical variations across individuals, we introduced a corrected version of Index A, termed the Hiki index (HI), by dividing Index A by the distance from the vertebral center to the left diaphragmatic angle. This adjustment significantly enhanced reproducibility and standardization of measurements. The HI maintained high diagnostic accuracy for a range of gastrointestinal symptoms, with the strongest predictive performance (AUC 0.77) observed for therapeutic intervention. These results indicate that HI is a practical and reliable index that can be universally applied in clinical settings to guide postoperative management.

Third, validation set analysis confirmed the robustness and external applicability of the HI. Based on ROC analysis in the validation set, the optimal cutoff values for individual symptoms—such as nausea (48.8%), bloating (52.2%), and belching (53.0%)—were closely aligned with the empirically defined threshold of HI ≥ 51%. Although vomiting did not show a significant association, the high predictive accuracy for other symptoms supports the appropriateness of 51% as a unified, clinically applicable cutoff value for defining gastric distension. One methodological improvement in the validation cohort was the partial standardization of imaging timing. While radiographs were still occasionally taken in response to symptom onset, efforts were made to acquire images at least 1 h after meals whenever feasible. This approach likely reduced variability in gastric content conditions and enhanced the reproducibility of HI measurements. This partial standardization may partly explain the improved consistency in cutoff values and the robust performance of the HI across multiple symptoms in the validation cohort.

Interestingly, HI was not significantly related to other postoperative complications such as ileus or intra‐abdominal infections, suggesting that the index specifically reflects functional gastric stasis rather than general surgical morbidity. In addition, there were no significant differences in preoperative serum albumin levels, diabetes mellitus prevalence, or ASA physical status between patients with and without gastric dilatation. These findings suggest that these systemic factors were unlikely to have contributed substantially to postoperative gastric stasis in our study population. This specificity enhances the clinical value of HI as a focused tool for identifying patients at risk of gastric content retention following gastrectomy.

Furthermore, multivariable analysis identified age under 75 years, female sex, and undergoing PPG as independent risk factors for postoperative gastric dilatation. These findings are consistent with previous reports linking these demographic and procedural factors to delayed gastric emptying and dysmotility [13, 14]. Although not reaching statistical significance, other surgical factors such as open approach and extensive lymphadenectomy also showed relatively higher odds ratios, suggesting a possible contribution of surgical invasiveness to postoperative motility impairment.

In addition to patient demographics, our analysis identified a significant association between the type of surgical procedure and the occurrence of gastric distension. Specifically, PPG was more frequently observed in the distension group, suggesting that preservation of the pyloric ring may contribute to delayed gastric emptying and functional distension of the remnant stomach. Interestingly, despite the intention of preserving pyloric function through hepatic branch and pyloric cuff preservation in PPG, gastric distension was more frequent in this group. This paradox may be explained by the preserved pylorus functioning as a transient outflow resistance postoperatively, contributing to delayed gastric emptying despite intact neural pathways. Although this may act as a confounding factor, it also highlights the importance of considering procedural differences in the interpretation of HI‐based gastric stasis.

To further investigate whether anatomical variations influence HI values, we analyzed the impact of esophageal hiatal hernia using preoperative endoscopic data. Although univariate analysis showed a significant difference in the prevalence of hiatal hernia between groups, multivariate analysis did not identify hiatal hernia as a significant factor. Therefore, the presence of a minor hiatal hernia is unlikely to contribute meaningfully to increased gastric dilatation in this cohort. Further studies using detailed imaging‐based assessments may help clarify the role of such anatomical variations.

We recognize that both radiographic gastric distension and gastrointestinal symptoms likely reflect the same underlying physiological process—delayed gastric emptying. However, our aim in correlating these findings was not to propose a novel pathophysiological mechanism, but rather to assess whether an objective radiographic marker, such as the HI, could serve as a surrogate for clinically significant stasis. In postoperative care, subjective symptoms are often inconsistently reported and may be underrecognized in patients with impaired communication or frailty. The HI provides a reproducible and practical imaging‐based metric that may assist clinicians in identifying at‐risk patients and initiating timely interventions, regardless of subjective symptom reporting. Early identification of gastric stasis using the HI may support clinical decision‐making aimed at reducing the risk of complications such as vomiting‐related aspiration. Although such events are relatively uncommon after gastrectomy, risk stratification using an objective index may contribute to safer postoperative care.

Furthermore, although therapeutic interventions were not included as statistical endpoints in the validation cohort, institutional practice since the implementation of the HI in 2022 required intervention (e.g., fasting, decompression, or pharmacologic therapy) when the HI exceeded 51%, regardless of symptoms. Therefore, the observed association between HI and gastrointestinal symptoms in the validation cohort should be interpreted as an exploratory correlation rather than a direct reflection of clinical decision‐making.

This study has several limitations. First, this study (In particular, regarding the test set) was conducted retrospectively, and observational biases or inconsistencies in data collection cannot be entirely excluded. Second, the decision to initiate therapeutic intervention was made at the discretion of the attending surgical team, based on clinical symptoms, physical findings, and radiographic interpretation. Therefore, we cannot fully exclude the possibility that the presence of radiographic gastric dilatation influenced treatment decisions, such as fasting or nasogastric tube placement. This raises the potential for tautological bias, where the predictor (HI) may have contributed to the outcome (intervention), thereby confounding the observed association. While the significant correlation between the HI and intervention supports its potential utility, this relationship must be interpreted with caution. Future prospective studies should implement standardized intervention criteria to eliminate this circularity and more rigorously validate the clinical predictive value of the HI. Third, although the validation set was included, this study was limited to a single institution, and further multicenter collaborative research is needed for external validation. Fourth, the timing of X‐ray imaging (e.g., postprandial or fasting state) was not fully standardized, potentially introducing variability in the measured indices. Furthermore, in cases where the stomach is excessively filled with fluid or food content postoperatively, the gastric bubble may be absent or difficult to delineate on plain abdominal radiographs. In such cases, the HI cannot be reliably measured, limiting its applicability. This constraint highlights the need for alternative imaging or assessment strategies in patients with complete gastric content retention.

In, conclusion, this study established the HI as a novel, objective, and reproducible radiographic metric for assessing postoperative gastric dilatation in gastrectomy patients. The HI demonstrated high diagnostic accuracy for predicting gastrointestinal symptoms and the need for therapeutic intervention, providing a standardized approach to identify patients at risk of gastric stasis. The use of HI may improve postoperative care by facilitating early intervention, optimizing symptom management, and enhancing patient outcomes. However, further multicenter studies are needed to confirm its generalizability and clinical utility in diverse patient populations.

## Author Contributions

**Hiroki Harada:** conceptualization, methodology, data curation, investigation, formal analysis, validation, visualization, writing – original draft, writing – review and editing, project administration. **Yoshiko Yamaoka:** methodology, investigation, validation, writing – review and editing. **Akiko Watanabe:** conceptualization, methodology, data curation, investigation, formal analysis, writing – review and editing. **Kota Okuno:** data curation, software, writing – review and editing, formal analysis. **Shohei Fujita:** investigation, writing – review and editing, data curation. **Mikiko Sakuraya:** investigation, validation. **Tadashi Higuchi:** writing – review and editing, supervision, resources. **Koshi Kumagai:** writing – review and editing, supervision, visualization. **Keishi Yamashita:** conceptualization, methodology, supervision, writing – original draft, writing – review and editing. **Naoki Hiki:** conceptualization, methodology, supervision, writing – original draft, writing – review and editing.

## Ethics Statement

All procedures were conducted in accordance with the ethical standards of the responsible institutional and national committees on human experimentation and with the 1964 Helsinki Declaration and its later amendments. This study was approved by the Institutional Review Board of Kitasato University (Approval No. B19‐280).

## Consent

Informed consent, or a substitute for it, was obtained from all patients for inclusion in this study.

## Conflicts of Interest

Drs. Hiroki Harada, Keishi Yamashita, and Naoki Hiki serve as Editorial Board members for the Annals of Gastroenterological Surgery. Koshi Kumagai has financial relationships involving honoraria from Abbott Japan LLC, The Japan Surgical Society, Medtronic Japan Co. Ltd., Miyarisan Pharmaceutical Co. Ltd., Nobelpharma Co. Ltd., Nutri Co. Ltd., Zeon Medical Inc., and Zeria Pharmaceutical Co. Ltd. outside the submitted work.

## Supporting information


**Table S1:** Association between preoperative clinical characteristics and the Hiki Index.
